# The application of artificial gravity in medicine and space

**DOI:** 10.3389/fphys.2022.952723

**Published:** 2022-08-29

**Authors:** Eugenia Isasi, Maria E. Isasi, Jack J. W. A. van Loon

**Affiliations:** ^1^ Centro de Terapia Gravitacional, Montevideo, Uruguay; ^2^ Departamento de Histología y Embriología, Facultad de Medicina, Universidad de la República, Montevideo, Uruguay; ^3^ Department of Oral and Maxillofacial Surgery/Pathology, Amsterdam Movement Sciences & Amsterdam Bone Center (ABC), Amsterdam UMC location Vrije Universiteit Amsterdam & Academic Center for Dentistry Amsterdam (ACTA), Amsterdam, Netherlands; ^4^ Life Support and Physical Sciences Section (TEC-MMG), European Space Agency (ESA), European Space Research and Technology Centre (ESTEC), Noordwijk, Netherlands

**Keywords:** human centrifugation, microgravity, Peripheral Artery Disease (PAD), Coronary Artery Disease (CAD), Lymphedema, Complex Regional Pain Syndrome (CRPS), Secondary Raynaud’s Phenomenon, Systemic Sclerosis

## Abstract

Gravity plays a crucial role in physiology. The lack of gravity, like in long duration spaceflight missions, cause pathologies in *e.g.*, the musculoskeletal system, cardiovascular deconditioning, immune system deprivation or brain abnormalities, to just mention a few. The application of artificial gravity through short-arm human centrifugation (SAHC) has been studied as a possible countermeasure to treat spaceflight deconditioning. However, hypergravity protocols applied by using SAHC have also been used to treat different, ground-based pathologies. Such gravitational therapies have been applied in Uruguay for more than four decades now. The aim of this overview is to summarize the most important findings about the effects of gravitational therapy in different, mainly vascular based pathologies according to the experience in the Gravitational Therapy Center and to discuss the current research in the field of hypergravity applications in medicine but also as multisystem countermeasure for near weightlessness pathologies. New insight is needed on the use of hypergravity in medicine and space research and application.

## 1 Introduction

Gravity (g) plays a vital role in physiology. Near weightlessness in long duration spaceflight, causes pathologies such as severe loss of bone density and skeletal muscle strength, cardiovascular deconditioning, immune system deprivation, changes in brain morphology, reduced cognition or changes in vision (Paez et al., 2020; Roberts et al., 2017; Stepanek et al., 2019). Increased gravity (hyper-g) on the other hand is experienced by pilots of high-performance aircraft who are trained to undergo short periods of 8–9 g using dedicated training centrifuges. Centrifuges generating artificial gravity (AG) have been and still are being explored as multi-system countermeasure/therapeutic devices for the earlier mentioned spaceflight related pathologies for future long-duration spaceflights. However, AG exposure through SAHC has been used as a therapeutic procedure called Gravitational Therapy (GT) in Uruguay for more than 40 years now. It has been applied for the treatment of different vascular based pathologies such as peripheral obstructive arteriopathies, coronary artery disease, lymphedema, Raynaud phenomenon, among others with very successful results (Isasi et al., 1986a; Isasi et al., 1986b; Isasi et al., 1990a; Isasi et al., 2001). Besides, SAHC was also used as a treatment for obstructive peripheral arteriopathies in a Russian facility at Samara State University (Makarov and Lukashou, 2018) and more recently as a promising physical rehabilitation approach for other medical conditions (Kourtidou-Papadeli et al., 2021a; Kourtidou-Papadeli et al., 2021b). The aim of this overview is to summarize the most important findings about the therapeutic effects of GT through SAHC in different vascular based pathologies mainly according to the experience in the Gravitational Therapy Center in Uruguay and to discuss the current research in the field of AG applications in space research.

## 2 Hypergravity in medicine and space flight

It was the Dutch mathematician Christiaan Huygens who first introduced the term “centrifugal force” in his 1659 work “*De Vi Centrifuga”*. In human research and application, one of the very early predecessors of the current short arm centrifuges was probably the Cox’s chair described more than two centuries ago in his book “*Practical Observations on Insanity*” (Wade, 2005). Also the later work by Halloran and his application of a circulating swing used in clinical medicine in treatment of mental health issues laid the ground works for rotating devices in human medicine (Breathnach, 2010).

### 2.1 History of gravitational therapy in Uruguay

**FIGURE 1:**
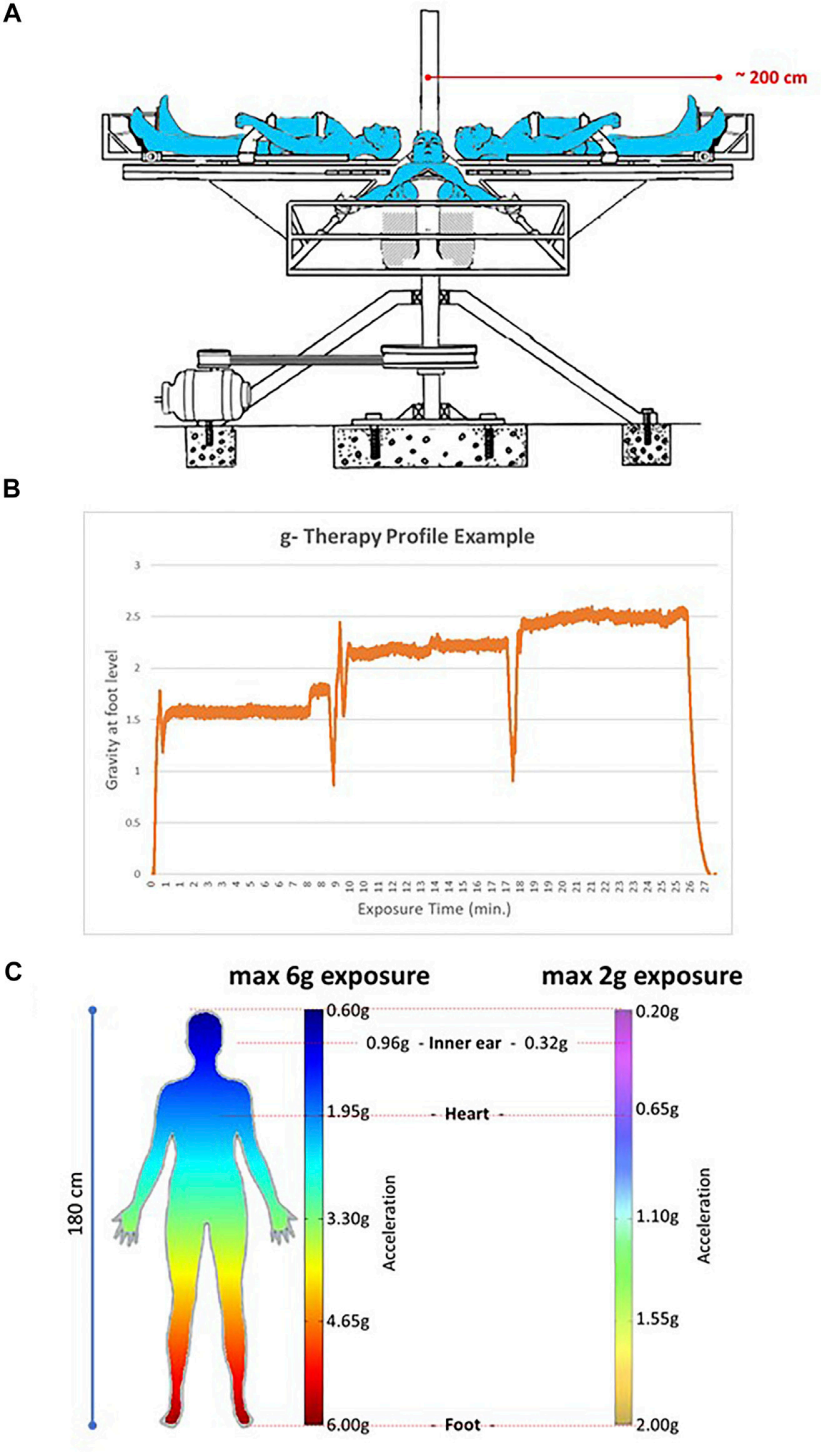
Centrifuge as currently used at the Gravity Therapy Center (GTC) in Montevideo, Uruguay. **(A)** Configuration of the nearly 2-m diameter centrifuge used for GT sessions at the GTC. A maximum of 4 patients can be accommodated in one session. **(B)** An example of a g profile used nowadays. The profile might be adapted based on patient’s tolerance, medical condition or age. **(C)** Gravity gradient over the patient’s 180 cm tall body when exposed to a maximum of 6 g or 2 g in a 2-m diameter system. Note that for the 6 g system the g at heart level is ∼1.95 g and at the level of the inner ear/vestibular system nearly 0.96 g. For the 2 g this is 0.65 and 0.32, respectively.

In late 1940s cardiologists Dr. E.J. Isasi and Dr. R. Velasco Lombardini built a first human centrifuge in Uruguay with the aim to explore a possible physical procedure to reduce arterial hypertension. This application of AG was inspired by works emerging from the Royal Canadian Air Force in Toronto published by Franks, Kerr & Rose (Franks et al., 1945a; Franks et al., 1945b). At that period in time there was no medication to treat this condition, as it was before the widespread use of *Rauwolfia* derivatives, vasodilators (hydralazine) and peripheral sympathetic inhibitors (guanethidine) (see review (Moser, 2006)). Some preliminary successful results on patients with arterial hypertension and peripheral arteriopathy disease (PAD) were communicated in national scientific meetings in the mid-1950s (Velasco-Lombardini and Isasi, 1951). The centrifuge was no longer used until mid-1970s when cardiologists Dr. M.E. Isasi and Dr. E.S. Isasi began to explore the effects of AG in different cardiovascular pathologies. In 1979 they devised a new centrifuge that was later patented in Uruguay, Argentina and United States (Figure 1A). With this new system they founded the Gravitational Therapy Center (GTC) in Montevideo, Uruguay, in that same year. To date the GTC still receives patients referred from different medical institutions for the treatment of various vascular based pathologies.

### 2.2 GT protocol

M.E. Isasi and E.S. Isasi have developed hypergravity protocols in which subjects are placed on a human centrifuge (US patent 4,890,629) in supine position with the head towards the axis of rotation (Figure 1A) and are exposed to accelerative and decelerative profiles (+Gz) from 0 to a maximum of 6 g at foot level with a rapid onset to the peak of acceleration and a rapid ramp back (Figure 1B) (Isasi et al., 2001; Isasi et al., 1986a; Isasi et al., 1986b; Isasi et al., 1992a; Isasi et al., 1992b; Isasi and Isasi, 1992; Isasi et al., 1992c; Isasi and Isasi, 2007; Isasi et al., 2007; Isasi et al., 2014).

By this procedure a gradient of g levels is imposed from head to feet (Figure 1C) (Isasi et al., 2014) although in the very first centrifuge and protocols patients were exposed seated, as was based on aerospace pilot centrifuge protocols. In some protocols g profiles could reach up to 6 g at foot level during seconds in trained patients. Nowadays, most protocols applied use g profiles between 1.5 and ∼2.5 g at foot level during longer periods in a total of 1-h treatment. These lower g levels are very well tolerated in most patients of a broad age range from both sexes and with beneficial therapeutic effects (Figure 1B). After some training sessions, patients receive in average 1-h session, 1–3 times/week to complete at least 20 sessions. Importantly, the number of sessions and the maximum g level reached may vary according to each patient condition (*e.g.*: age, medical condition, tolerance) (Isasi et al., 1990a; Isasi et al., 2007; Isasi et al., 1992a; Isasi et al., 1992b; Isasi and Isasi, 1992).

By means of safety belts, patients are strapped with their head close to the center of a nearly 2 m-radius centrifuge. Patients are provided with an eye cover and they are asked to avoid moving their heads during centrifugation to avoid Coriolis effects. The clinical assessment and arterial blood pressure measurement is always performed before and after GT protocol. Currently, ambulatory electrocardiogram and blood pressure monitoring during centrifugation are also used. A physician and assistant are always monitoring patients during centrifugation in case any patient requests to stop centrifugation by raising the hand or speaking out loud. Over more than 40 years, GT has demonstrated to be a safe and well tolerated therapeutic procedure, without significant side effects in the conditions applied. There were only sporadic transitory states of dizziness or nausea observed when the patient is not yet used to the procedure, so, the training of patients is important for better tolerance and continuity of the treatment.

### 2.3 Decades of studies on the effects of centrifugation on different pathologies

#### 2.3.1 Peripheral artery disease (PAD)

**FIGURE 2:**
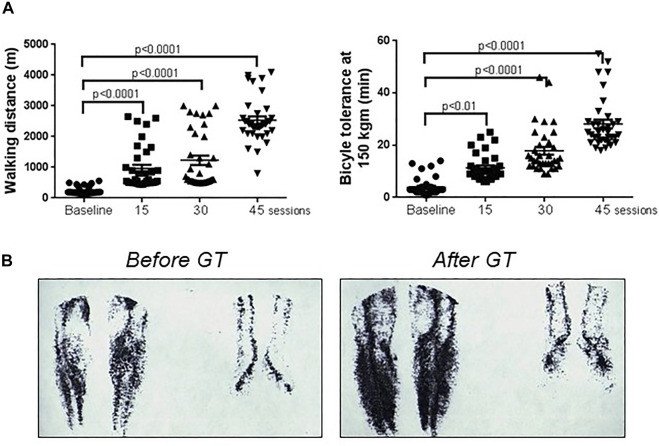
Evolution of patients with peripheral artery disease with gravitational therapy. **(A)** Functional recovery of 35 PAD patients with intermittent claudication (Fontaine stage II) evaluated through total walking distance (left chart) and bicycle tolerance at 150 kgm/min (right chart) following GT sessions. Note the significant improvement in both parameters evidencing functional recovery following GT sessions. **(B)** Static radionuclide angiography of calves and feet of a 57-year-old male patient with PAD before (left image) and after GT rehabilitation (45 sessions) (right image). Note the increase of blood circulation in the lower limbs. *Adapted from* (Isasi et al., 1990a)*.*

By continuing the initial observations on the effects of centrifugation on PAD patients a study to rehabilitate patients with peripheral obstructive arteriopathies was initiated. 35 patients with intermittent claudication (Fontaine stage II) were exposed to hypergravity protocol during 30 min, 3 times/week during 20 weeks. Functional recovery was measured by the total walking distance and the duration of bicycle tolerance at 150 kgm/min at baseline and after 15, 30 and 45 hypergravity sessions (Isasi et al., 1986a). A very significant improvement in functional capacity was observed with the greater number of hypergravity sessions (Figure 2A). Interestingly, static radionuclide angiography of thighs and calves performed before and at the end of the study revealed a greater blood circulation in the lower limbs of PAD patients which correlated with the functional recovery (Figure 2B). In another study of 10 male patients with PAD, a daily protocol of GT was applied during 30 min along 4 weeks. A significant increase in walking distance, sural triceps test score and reduced ischemic pain was observed after 4 weeks (Isasi et al., 1990b). Thus, GT improved PAD patients by abolishing muscle ischemic pain and recovering the functional capacity due to an increase in the collateral circulation (Isasi et al., 1986a; Isasi et al., 1990b).

#### 2.3.2 Coronary artery disease (CAD)

GT has also been studied in patients with CAD since mid-1970s, showing a significant improvement in functional capacity and in exercise tolerance as well as reduced angina pectoris (Isasi et al., 2000a). In a study of 20 patients with CAD, with mild or moderate hypertension, with or without angina pectoris and with ST depression >0.2 mV, exercise bicycle testing was assessed before and after one GT session. The treatment resulted in a significant decrease in ST depression and increase in exercise duration without angina pectoris (Isasi et al., 2000a). This acute effect following one hypergravity exposure might indicate increased myocardial perfusion. In another study of 30 patients with proven CAD and 10 healthy volunteers (HV), endothelial cells (ECs) were counted in venous blood smears stained with May-Grünwald–Giemsa over 100 white blood cells (WBC) obtained before and after only one GT session. Very low or undetectable ECs were observed in the control group at baseline and after one hypergravity protocol (ECs/100 WBC: from 0.4 ± 0.5 to 0.7 ± 0.6). In turn, CAD patients showed a significant increase in ECs (ECs/100 WBC: from 4.27 ± 4.09 to 11.5 ± 7.55) (Isasi et al., 1998a). In addition, CAD patients did also show a statistically very significant increase in ECs number with respect to HV at baseline which is consistent with the literature that show increased number of circulating ECs associated with cardiovascular disease and its risk factors, such as unstable angina, acute myocardial infarction, stroke, diabetes mellitus, critical limb ischemia and related diseases (Boos et al., 2006; Deb-Chatterji et al., 2020; Schmidt et al., 2015). Thus, taken the results on PAD and CAD patients, it is feasible that GT would induce sustained vasodilation, increase endothelium turnover and collateral circulation.

#### 2.3.3 Lymphedema

In the 1980s the GTC began a study on the possible effect of centrifugation on lymphedema patients (Isasi et al., 1986b). Lymphedema is a progressive pathological condition of the lymphatic system that involves the accumulation of protein-rich fluid, inflammation, swelling and subsequent induration and fibrosis that cause disfigurement of the affected region, decreased mobility and function (for review see (Warren et al., 2007) and (O'Donnell et al., 2020)). In a study of 30 patients with primary or secondary lymphedema due to surgical procedures, irradiation therapy, erysipelas infection or chronic venous insufficiency affecting only one lower limb (26 pts, 87%) or both lower limbs (4 pts, 13%) and 10 HV received a subcutaneous injection of 1–1.5 mCi of 99mTc Sb2S3 colloid into the foot first interdigital space. Gamma camera images (5 min-static images) were taken from the injection site and the abdomen immediately after and at 1, 2, 3 or 4 hs post-radionuclide injection in patients in supine position (Isasi et al., 1992a). In 1 g control conditions, the radionuclide liver uptake occurred at 3 hs in 10 (100%) HV and 26 (87%) lymphedema patients and at 4 hs in 4 (13%) lymphedema patients (Isasi et al., 1992a). In a second session using the same protocol but now including a hypergravity protocol (accelerative and decelerative profiles, 0–6, + Gz) exposure of 20 min, showed that in 10 (100%) HV and 28 (93%) lymphedema patients radionuclide liver uptake was observed already at 1h, in 1 (3%) patient at 2hs and in 1 (3%) patient at 3hs after tracer injection (Isasi et al., 1986b). Interestingly, in another study of 20 patients (3M, 17F, mean age 50 yrs old) and 10 HV (3M, 7F, mean age space, 48 yrs) two lymphoscintigraphic studies were performed at baseline and after centrifugation, without medication or after taking cyclooxygenase (COX) inhibitors (aspirin 500mg/8hs, indomethacin 25 mg/8 hs) starting 48hs prior to the study. Results showed that COX inhibitors delayed (2–4+ hs) the radionuclide liver uptake significantly in HV and lymphedema patients while without medication the liver uptake was observed at 1 h in 10 HV and 18 patients and 2 h+ in 2 patients after centrifugation (Isasi et al., 1990a; Isasi et al., 1992c). Thus, the hypergravity protocol applied constitutes a mechanical stimulus that accelerated the lymphatic drainage and the liver uptake of tracer in HV and patients and involved a mechanism related to, at least in part, prostaglandin synthesis (Isasi et al., 1990a; Isasi et al., 1992c). The relationship between endothelium derived prostanoids and the modulation of lymphatic tone, flow resistance, and lymphatic function has been documented in other papers (Gashev and Zawieja, 2010; Koller et al., 1999).

**FIGURE 3:**
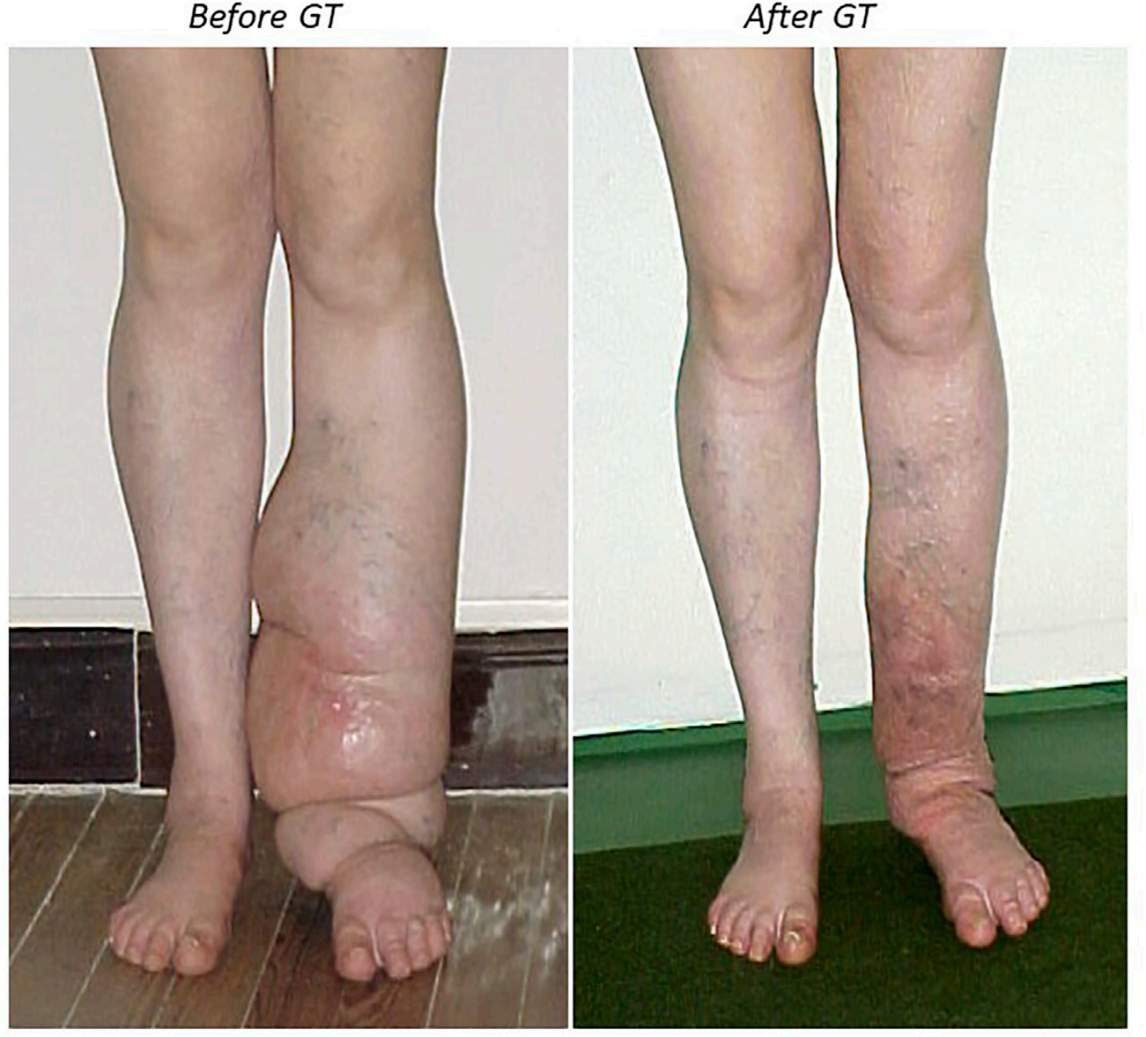
A lymphedema patient with gravitational therapy. A 48-year-old female patient with a severe lymphedema affecting left lower limb after 13 years of numerous erysipelas episodes (left). After 5 weeks of initiating gravitational therapy (10 sessions) there was a significant reduced edema (right). She could wear trousers and could sleep without the limb elevated. The leg perimeter had reduced from 70 to 29 cm, the quality of the skin improved and she did not repeat erysipelas.

Patients with Primary or Secondary Lymphedema treated with GT showed reduced edema, improved mobility, reduced the frequency of erysipelas episodes, improved the quality of the skin and the sensitivity of the lymphedematous limbs (Isasi et al., 2001; Isasi and Isasi, 2007) (Figure 3). In a study that recorded the evolution of 275 lymphedematous limbs from 161 patients (138 F, mean age 50.6 ± 18 yrs and 23 M, mean age 42 ± 20 yrs) suffering from Primary or Secondary Lymphedema that were treated with an average of 20 GT sessions (two to three sessions/week) and a maintenance therapy of a minimum of 10 sessions annually, showed that 195 pts (71%) reduced more than 50% of the lymphedematous limb volume, 56 pts (20%) between 30–50% of the volume and 24 pts (9%) less than 30% of the limb volume (Isasi and Isasi, 2007). After GT, adequate elastic support maintained the successful results. Although lymphedema cannot be completely cured, GT has demonstrated to significantly reduce edema and functional disability of the limb, improving the quality of life of these patients.

#### 2.3.4 Complex regional pain syndrome (CRPS)

GT also has beneficial therapeutic effects in patients suffering from Complex Regional Pain Syndrome (CRPS) (Isasi et al., 1990c; Isasi et al., 1999). CRPS type I, also formerly known as reflex sympathetic dystrophy (RSD), has been described as pain that a patient feels which is disproportionate to the inciting event and is associated with autonomic dysfunction, swelling, dystrophic skin changes, stiffness, functional impairment and eventual atrophy (Neumeister and Romanelli, 2020). This disease affects musculoskeletal, neural and vascular structures and may be presented as continuing pain, allodynia or hyperalgesia, changes in skin perfusion or abnormal sudomotor activity and also thermoregulatory dysfunction with distinct vascular dysregulation patterns (Neumeister and Romanelli, 2020). In fact, changes in vascular sensitivity to cold and circulating catecholamines may be responsible for vascular abnormalities and may be associated with an abnormal (site dependent) reflex pattern of sympathetic vasoconstrictor neurons due to thermoregulatory and emotional stimuli (Baron and Maier, 1996). In a study of 26 patients (5M, 21F, mean age 42 yrs old) suffering from RSD triggered by trauma (22 pts) or by surgery (4 pts), patients were exposed to centrifugation (+Gz, 0–6g, 1 h) daily. The number of sessions was dependent on patient’s response (from 2 to 20 sessions). Assessment of patients was performed by clinical evaluation, digital photoplethysmography and static radionuclide bone images. In 25 patients (96%) there was a dramatic pain relief and improvement in vascular response. Radionuclide bone images persisted positive in spite of the clinical improvement and good vascular response (Isasi et al., 1990c). Importantly, GT seems to be an effective therapy in the acute but not in the chronic stage of CRPS I (Isasi et al., 1999).

#### 2.3.5 Secondary Raynaud’s phenomenon and systemic sclerosis

**FIGURE 4:**
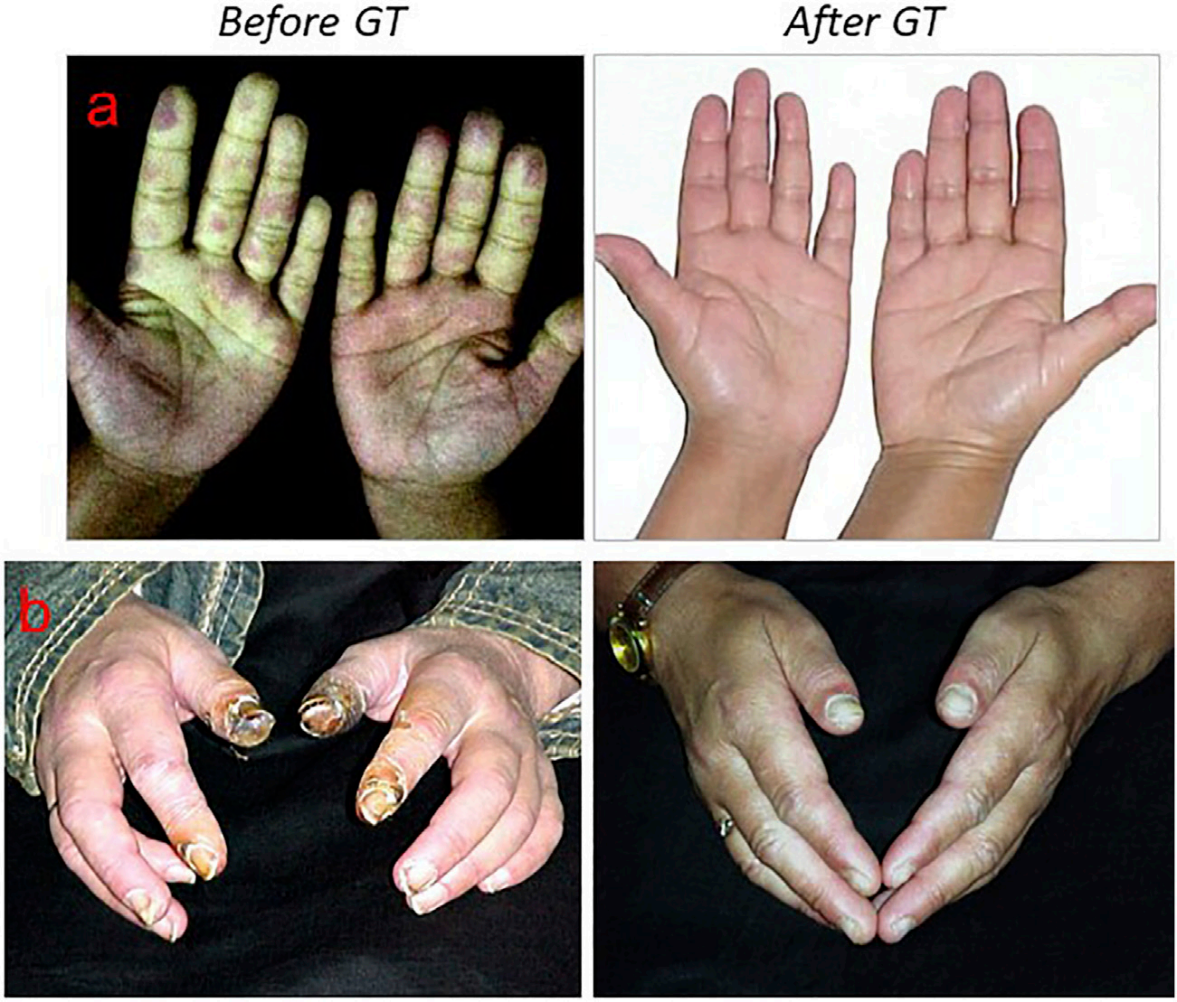
Two examples of patients with severe secondary Raynaud´s phenomenon treated with gravitational therapy. **(A)** A 36-year-old female patient with severe secondary Raynaud’s Phenomenon. Before treatment, severe vasoconstriction (pallor and cyanosis) and puffy fingers (left image). After gravitational therapy patient showed a remarkable improvement in blood circulation of both hands (right image). **(B)** A 44-year-old female patient diagnosed with mixed connective tissue disease that presented a 10-days- history of painful fingertips ischemia and necrosis in both hands (left image). The ischemic pain was so severe that kept her awake all night long. After one week of gravitational therapy pain reduced dramatically and she did not take analgesics any longer. After 20 weeks of gravitational therapy the fingers were completely healed (right image).

Secondary Raynaud´s Phenomenon (RP) is associated with Systemic Sclerosis (SSc) and it is a vasoconstrictive response associated to endothelial dysfunction that is present in approximately 95% of patients with SSc (Meier et al., 2012). SSc is a progressive autoimmune connective tissue disease characterized by vascular abnormalities and fibrosis of the skin, joints and internal organs (Cutolo et al., 2010; Denton and Khanna, 2017; Quinlivan et al., 2020). Given that microvascular damage and endothelial dysfunction underlies SSc and that GT has shown beneficial effects on vascular diseases by inducing sustained vasodilation through prostacyclin release and by increasing endothelium turnover (Isasi et al., 1998a), GT was postulated to be beneficial in SSc patients. In a study of 46 patients (6 F, 40 M, mean age 48 ± 14 yrs old) with SSc that received GT, 3 times a week during 6 weeks, patients showed increased pulse wave amplitude and very significant increased ECs in venous blood smears after GT. Also, SSc patients showed a very good clinical evolution by reducing ischemic pain, less frequent Raynaud´s attacks and by healing digital ulcers avoiding, in severe cases, the digital amputation (Figure 4) (Isasi and Isasi, 2004). In this study the follow-up period was 37 ± 14 months and all patients received between 10 and 20 hypergravity sessions prior to winter as a maintenance therapy. With respect to skin fibrosis, SSc patients showed a clear improvement after GT. In a former study with 20 SSc female patients that received GT, skin sclerosis was assessed by the % of skin involvement applying the “rule of nines” as in burns and by a clinical skin severity core (CSSS) rating the thickness of the skin from 0 to 3+ in 15 areas of the body. In this study, the mean skin scores at the entry versus the end of GT were 50.21 vs. 19.80 for % of skin involvement and 22.72 vs. 9.16 for CSSS, showing an improvement in skin fibrosis (Isasi et al., 1998b). In addition to the beneficial effects reported on RP, digital ischemic lesions and skin fibrosis of SSc patients (Isasi et al., 1998b; Isasi et al., 2000b; Isasi and Isasi, 2004), digestive symptoms, mouth opening, mild esophagitis and esophageal hypomotility, did also improved after GT (Isasi et al., 2006). In addition to SSc patients, patients with localized scleroderma (morphea) or linear scleroderma did also show a significant improvement with GT. Six patients with linear scleroderma of upper or lower limbs (4F, 2M, mean age 16.0 ± 7.8 yrs old) that received GT over 3 months, showed a significant improvement in skin score, reducing sclerosis, regaining muscle strength and mobility of the affected limb (Isasi et al., 2007). In a more recent work that communicated the evolution of 90 patient (76F, 14M, mean age 50 ± 14 yrs old) with RP and SSc that had digital ischemic lesions (150 fingers with digital ulcers and 53 with critical ischemia) recorded at the entrance of therapy, received GT 2–3 times/week during 6–8 weeks. After rehabilitation, most patients received a maintenance therapy of 10–20 sessions annually. Most patients (98%) with digital ischemic vascular pain achieved complete pain relief after two to three hypergravity sessions and could refrain from opioid analgesics. In addition, frequency of RP attacks and severity was reduced, improving skin color, temperature, sensitivity and mobility because of reduced edema in hands. At the beginning, 33 patients had 53 fingers with critical ischemia and after GT, 31 patients (94%) completely healed the ischemic lesions while 2 patients (6%) suffered distal digital amputation during treatment. No adverse effect occurred in any patient exposed to GT during the follow-up period of 4.56 ± 3.18 years (Isasi et al., 2014). This study showed that GT had significant beneficial effects preventing the use of aggressive treatment such as potent vasodilators, opioid analgesics and even hospitalization in cases with severe digital ischemic lesions. This mechanical/biophysical stimulation of the macro‐ and micro vasculature seems to be in line with a more recent study by Mitropoulos *et al.* (Mitropoulos et al., 2018) which suggest that 12 weeks twice a week of high intensity arm cranking training has the potential to improve the microvascular endothelial function in SSc patients. Also, our own work with ECs exposed to hypergravity (Szulcek et al., 2015) and work by others (Maier et al., 2015) show a direct effect of gravity on ECs.

### 2.4 Biological effects of GT

Applying a GT protocol showed a vasodilator effect assessed with digital photoplethysmography at 5, 15, 30 and 60 min after centrifugation in patients and HV and it was abrogated with the use of COX inhibitors such as aspirin (500 mg/8 hs) or indomethacin (25 mg/8 hs) starting 72 hs prior to centrifugation (Isasi and Isasi, 1992; Isasi and Isasi, 1996). These initial results obtained led to study levels of 6-keto prostaglandin F1 alpha, a prostacyclin active and stable metabolite, in venous blood samples of 30 patients with coronary artery disease (CAD) and 4 controls. In control subjects this resulted in a statistically significant increase from 241 ± 50 pg/ml to 535 ± 76 pg/ml and in CAD patients from 167 ± 43 to 359 ± 79 pg/ml after only one GT session of 1 h (Isasi et al., 1998a). This suggests that the hypergravity protocol would induce a vasodilator effect dependent on prostacyclin synthesis and release. In addition, GT would have other important biological effects considering the multiple properties of prostacyclin such as the inhibition of platelet aggregation or white blood cells adhesion (Moncada, 2006). On the other side, data from basic spaceflight related research suggest that SAHC providing mild hyper‐g (1–2 +Gz) results in a gravitational‐induced rise of transmural pressure leading to constriction of resistance vessels that contribute to the maintenance of the mean arterial pressure as well as microvascular blood pooling contributing to soft tissue capacitance in the lower extremities (Watenpaugh et al., 2004; Habazettl et al., 2016). Although this acute effect of vasoconstriction in the lower extremities occurs during the acceleration phase at + Gz exposure due to high transmural pressures, in the deceleration and re-adaptation phases, the microvascular flow is recovered and enhanced as was demonstrated by digital photoplethysmography at different times after centrifugation (Isasi and Isasi, 1996)**.** Importantly, given that the endothelium lining small and large blood vessels is an important tissue sensitive to *e.g.* shear stress/mechanical stimulations during the hyper-gravity and the re-adaptation phases of the treatment, it has been speculated that ECs might play a central role in the therapeutic effects observed (Isasi et al., 1998a). In this sense, it is possible that enhanced hemodynamic forces generated by GT would induce the synthesis and release of other biologically active substances such as nitric oxide (Isasi et al., 1998a), the increased expression of growth factors and cytokines and the modulation of endothelial gene expression in analogy with shear stress effects on the endothelium (Ando and Yamamoto, 2009; Davies, 2009).

Other possible mechanisms involved might be the process of vasomotion and underlying processes in small arteries. Vasomotion is modulated by changes in transmural pressures in small blood vessels (Gustafsson, 1993) but also lymph vessels show vasomotion related to lymph-induced transmural pressure and flow rate (Helden and Zhao, 2000; von der Weid, 2001). On a more molecular level, possible modulations of the tyrosine phosphorylation pathway involved in cellular signaling in arteriolar myogenic constriction (Murphy et al., 2002) might be relevant in the effects of GT. It might even be speculated, since the latter is also mentioned to be related to SSc, that protein tyrosine kinases might play a role in GT since they are known players in SSc disease pathogenesis (Sacchetti and Bottini, 2017).

### 2.5 Ground-based applications of short-arm human centrifugation with therapeutic benefits

There have been specific applications using centrifuges for the treatment of medical issues in the past but most seemed not to have developed into a more elaborate use. A case report from 1966 mention the use of a centrifuge at the Mayo Clinic (United States) to treat a retinal detachment. The patient was placed in different positions on the centrifuge and exposed to g levels between 2 and 2.7 g (Neault et al., 1966). Apparently, the Mayo Clinic had a human centrifuge available in that period so it is to be expected that it was also used for other treatments.

In another case report the centrifuge at NASA Ames (Moffett Field, United States) was used to relocate a bullet fragment in the brain of a 63-year-old male assault victim into a fixed position. A procedure was applied where the patient was positioned mainly towards the center of rotation and was exposed to a g profile with a maximum load of 6 g and the acceleration acting along the patient Gx, Gy and Gz axis being 5.1, 2.6 and 0.9 g, respectively. The total procedure lasted 58 s (Pelligra et al., 1970).

**FIGURE 5:**
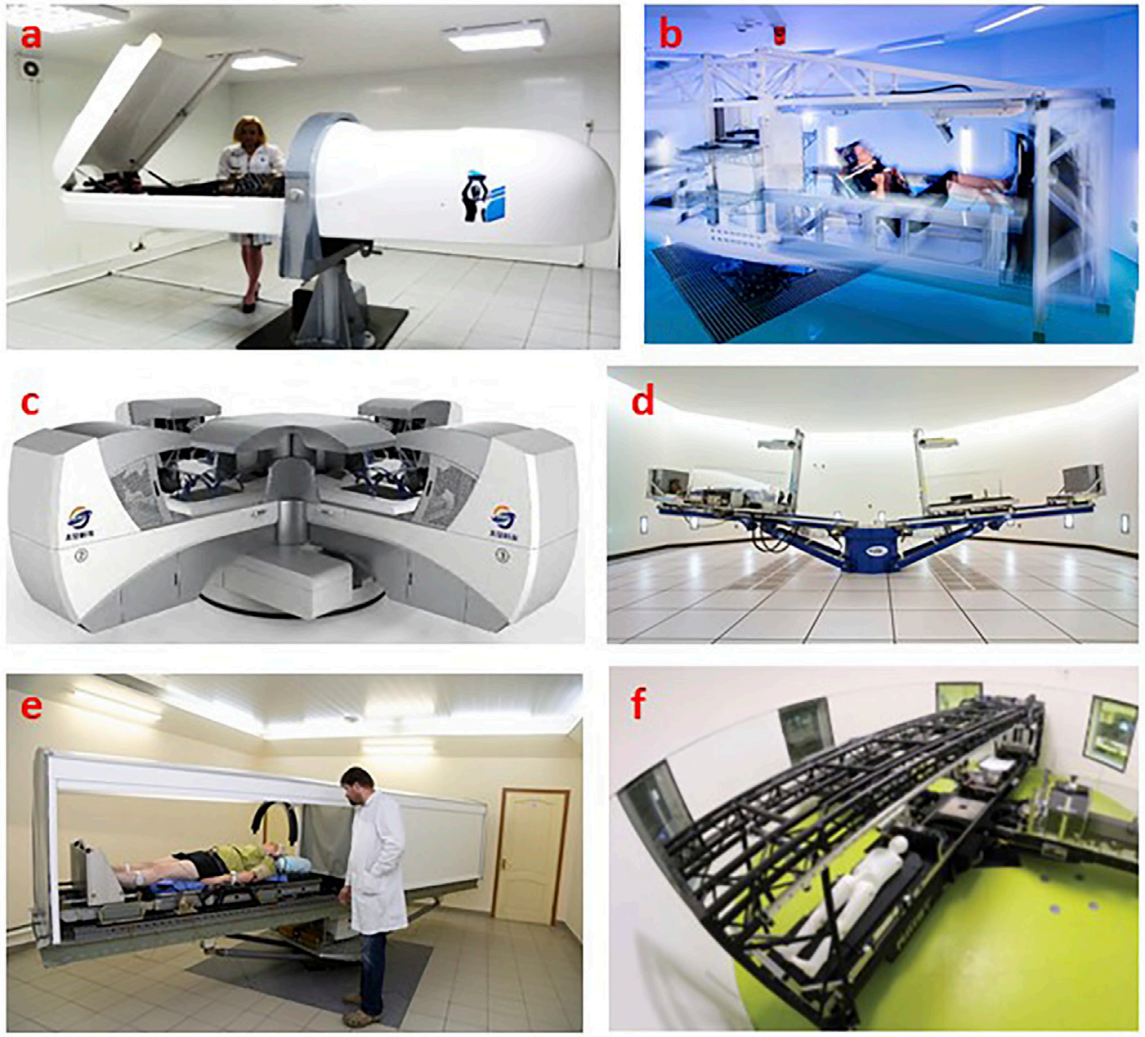
Some examples of short arm centrifuges used in treatment and in support of various space flight programs as countermeasure against microgravity related pathologies. **(A)** One-person gravity therapy centrifuge at the Samara State Medical University, Russia (Orlov and Koloteva, 2017). **(B)** Upgraded SAHC from European Space Agency, ESA, at the Olympic Sport Centre Planica, Slovenia (image ESA & Jozef Stefan Institute Slovenia/^©^K. Bidovec & A. Hodalič). **(C)** Centrifuge at: Department of Health Technology, Space Institute of Southern China, Shenzhen, Guangdong, China. **(D)** NASA short radius centrifuge currently being re-installed at Texas A&M University, United States (Arya et al., 2007). **(E)** Short arm centrifuge at the Institute for Biomedical Problems, IMBP, Moscow, Russia. **(F)** Human Centrifuge at the:envihab center of the German space agency, DLR near Cologne, Germany (Frett et al., 2014).

There are also reports from the Samara State Medical University (Samara, Russia) on the successful treatment of patients with chronic lower limb ischemia stage II with the support of a short arm centrifuge (Figure 5A) (Galkin et al., 2003; Makarov, 2003; Makarov and Lukashou, 2018). Unfortunately, most of these reports have not been published in international journals.

Very recently a SAHC has been proposed as a robust countermeasure to treat deconditioning and seems to be a promising physical rehabilitation approach towards the prevention of musculoskeletal decrement due to confinement and inactivity. Studies from Kourtidou-Papadeli *et al.* (Kourtidou-Papadeli et al., 2021a; Kourtidou-Papadeli et al., 2021b) show significant changes in heart rate variability, cardiac output, cardiac index, cardiac power and mean arterial pressure at different AG levels as compared to standing position by using a protocol with a +Gz force up to 2 g at foot level. Interestingly, SAHC applied as a 4-weeks training program including three weekly sessions of 30 min of intermittent centrifugation at 1.5–2 g in a 54-year-old male patient with multiple sclerosis (MS) showed significant improvement in cardiovascular parameters, muscle oxygen consumption and MS-related mobility disability and balance capacity (Kourtidou-Papadeli et al., 2021b).

### 2.6 Artificial gravity in space flight

That the lack of gravity during orbital space flight might not be compatible with human physiology was already recognized by the Russian mathematics teacher and space pioneer Konstantin Tsiolkovsky (1857–1935). He was probably one of the first to perform serious engineering work on human space travel and he drew the concept of generating AG by rotating the complete spacecraft where humans could maintain a proper physiology, and thus survive, in outer space. This concept was elaborated upon by the Slovenian army officer Herman Potočnik published in 1929 (Noordung, 1929) and the well-known torus-shaped station from von Braun is also a large-radius rotation space station, published in 1952 (see for overview of space stations (Logsdon and Butler, 1985)).

Current research regarding AG and its application in long duration space missions is very much focused on relatively short-radius centrifuges. The dimensions of such short-arm human centrifuges are driven by the limited diameter of current spacecrafts’ hull. See examples of such centrifuges in Figures 5B–F. In light of a possible multi-system countermeasure for microgravity related pathologies, numerous studies have been executed on such small systems. We will address the outcome of only a few of these findings:

The exposure of humans to a 5-days ground-based model for near weightlessness, −6° head-down tilt bed rest (HDBR), had a major impact on the heart function and geometry. A daily exposure of either 30 min continuous or 6 × 5 min intermitted at 1.0 + G_z_ at the heart in a 2.82 m radius centrifuge did not counteract the effects of simulated near weightlessness while chronic exposure to 1 g (being ambulatory) for 3 days after the HDBR protocol did reverse the effects (Caiani et al., 2014). Using the same AG protocol but now in a 60-days HDBR study, De Martino and colleagues wanted to explore the possible efficacy of AG to mitigate deterioration in standing balance and anticipatory postural adjustments of trunk muscles. All experimental groups had poorer balance performance in most of the parameters and delayed trunk muscle responses. However, there was mitigation of some aspects of postural control in intermittent 1.0 + G_z_ exposure (De Martino et al., 2021).

Muscle performance was explored in, again, the same AG protocol but now in a 5 days HDBR study and AG 1.0 g + G_z_ set at the subjects’ estimated center of mass. The AG protocols were not able to prevent the catabolic effects of HDBR upon muscle and bone. There was, however, a preservation of vertical jump performance by AG but this was likely caused by central nervous rather than by peripheral musculoskeletal effects (Rittweger et al., 2015).

From a 21-days bedrest study where the treatment group was exposed to a continuous 1 h/day of AG (2.5 g at foot level, 2.2 m diameter centrifuge: +G_z_ at heart level ∼0.97 g) it was concluded that AG had the potential to maintain the functional (torque-velocity relationships of the knee extensors and plantar flexors of the ankle), biochemical and structural (mRNA and histology of vastus lateralis and soleus muscles biopsies), homeostasis of skeletal muscle in the face of chronic unloading (Caiozzo et al., 2009).

Also, a 2 × 30 min/day AG could not prevent the decreases in exercise capacity after the 4 days HDBR in male subjects. The authors recognized some indications to reverse hypovolemia induced by bed rest while they also concluded that SAHC might eliminate the changes in autonomic cardiovascular control (Iwasaki et al., 2001).

AG of 1 g at the center of the mass for either 30 min continuous or 6 × 5 min/day during 5 days HDBR exposure showed no relevant changes in bone markers such as urine levels of collagen type I C-Telopeptide (CTX), N-Telopeptide (NTX) as well as deoxypyridinoline (DPD) or serum betaCTX concentrations. However, there were significant changes in sCD200 and sCD200R1 markers (Kos et al., 2014) although the latter molecules are not widely used as bone related markers.

Bed rest decreased plasma volume, supine and head up tilt stroke volume in both HDBR as well as AG groups (1 h, 1.0 + Gz at the heart/day). Although plasma volume was decreased in control and AG after bedrest, AG was able to mitigate effects of 3 weeks of simulated microgravity on orthostatic tolerance and aerobic power. They argue that positive effects of AG on peripheral vasculature and improved sympathetic responsiveness to orthostatic stress might be the underlying mechanisms (Stenger et al., 2012).

## 3 Discussion

Gravitational Therapy as applied in Uruguay over more than four decades now, has shown important therapeutic effects in different medical conditions and in a broad range of patients’ ages. Patients from both sexes showed significant improvement in medical conditions, although there are reported differences in cardiovascular response upon + Gz exposure (Masatli et al., 2018). However, there is no study assessing differences in the therapeutic effectiveness between males and females with similar ages and medical conditions. Importantly, there were no significant side effects recorded even in patients that had a follow-up period longer than 20 years that received 10–20 GT sessions annually. The side effects encountered were very sporadic and were mostly transitory states of dizziness/nausea or very rare case of vomiting mostly related to head movements during centrifugation, ergo Coriolis effects. Also, among our exclusion criteria for GT are patients with active cancer, with advanced stages of dementia, with generalized infection or erysipelas infections, pregnant women, etc.

GT has shown a vasodilator effect through prostacyclin release, improving tissue perfusion and probably inducing collateral circulation, all resulting in functional improvement in CAD and PAD patients. It also significantly improved lymphedema patients by fostering lymphatic drainage, improving the quality of the skin, mobility and function of the lymphedematous limb. GT had also important beneficial effects in Secondary RP and SSc reducing frequency of RP´s attacks, reducing ischemic vascular pain, improving tissue perfusion and reducing skin fibrosis. It is also remarkable to note the improvement of patients in the acute phase of CRPS I. Although there is a variability in the evolution of each patient that mostly depends on the years of evolution and stage of the disease, other possible comorbidities and the pharmacological treatment received (type and dose of drugs), the success of GT treatment is also dependent on the frequency and number of sessions along time. Even though most of the experience in Uruguay is focused on vascular based pathologies and the majority of its effects might be explained on the mechanical stimulus over the blood vessels and the cardiovascular system, other body systems such as the immune and nervous system might also be involved in the clinical improvements observed.

Besides, the beneficial therapeutic effects of GT in several pathologies (*e.g.* secondary RP, vasculitis, SSc, *etc.*), usually surpasses the effects of medication and the importance of GT in non-curable pathologies (*e.g.* SSc, lymphedema, among others) with limited conventional medical treatment options is remarkable. Also, the use of GT could limit or even eliminate the use of some pharmaceutics with important side effects (Balakumar et al., 2015; Mladěnka et al., 2018) especially when such drugs have to be chronically administered.

One of the major limitations for GT widespread use application is the need of optimization and standardization of hypergravity protocols adapted to each patient and its medical condition. Moreover, the design of randomized clinical trials comparing the effects of GT versus conventional medical treatment (usually pharmacological) are also needed to better identify the specific potential of GT aside from other medical interventions.

Interestingly, new research on the application of SAHC in other human pathologies such as MS is emerging (Kourtidou-Papadeli et al., 2021a; Kourtidou-Papadeli et al., 2021b) and supports the long-standing observations in Uruguay regarding the beneficial therapeutic effects of GT in multiple pathologies. Besides, results on the Russian facility at the Samara State University in PAD patients (Makarov and Lukashou, 2018) also seem to be in line with results obtained in the GTC since 1980s. More research, clinical trials and publications are needed in this field to achieve an international validation of the hypergravity treatment and its widespread use.

Although the list of SAHC spaceflight related studies as discussed earlier is by far complete, it illustrated that there is a remarkable part of the protocols applied and parameters measured where AG does not resolve the HDBR related deteriorations but, at best, mitigate them. In this short overview of SAHC as multi-system countermeasure for spaceflight related immobilization we solely included studies/data using only AG as single modulation and not studies including additional stimuli such as ergometers (Iwase, 2005; Wang et al., 2011) or jumping protocols (Dreiner et al., 2020). The addition of other exercise means in addition to AG, already indicates that current short duration short-arm centrifuge protocols by themselves are not sufficiently effective to counteract HDBR related changes.

Regarding AG research applied for future long duration space flight; current short duration studies are mostly focused on relatively fast changing organ systems such as the cardiovascular system, sensory-motor system or muscles. Nearly no work has been done to explore the effects of short arm centrifuges on *e.g.,* bone, brain (morphology as well as function), vision or skin where the space related pathologies are especially more pronounced after long duration exposures to near weightlessness (Tronnier et al., 2008; Vico et al., 2017; Roberts et al., 2019; Van Ombergen et al., 2019; Paez et al., 2020).

Although the current SAHC-AG protocols are not or not sufficiently effective; what are the reasons and alternatives for insufficient AG protocols as microgravity countermeasure? This could be the very limited exposure times; some 30–60 min/day in many protocols. The effect of a response is often directly related to the amplitude and duration of the applied stimulus. With a 30 min 2 g at foot level exposure one only applies 1.6% of the daily g-dose at heart level or only 3.2% with a 1 h/day exposure. One might argue that replacing a deleted biological/physiological stimulus with only a few percent of its original magnitude might not be sufficient to restore a physiological state. Also, as shown in pre-clinical studies, the vestibular system, for long seen as ‘only’ having a balance supporting function, is involved in vestibulo-autonomic regulatory mechanisms targeting other organ systems such as the cardiovascular (Gotoh et al., 2003), musculoskeletal (Luxa et al., 2013; Morita et al., 2020), or the temperature regulating system (Fuller et al., 2002). With an AG exposure of 2 g at foot level in a 2-m diameter centrifuge the g-load to the vestibular system is only 0.32 g (see Figure 1C). With a 30 min/day exposure this generates a 0.7% stimulus compared to regular physiological conditions. It is plausible to presume that the central nervous system does not receive sufficient afferent signals from vestibular otoconia to perform its regulating function. However, we have to recognize also short-term high impact mechanical loads are sometimes sufficient to maintain proper physiology (Buckner et al., 2020; Lambert et al., 2020).

From the hypergravity therapies as developed and applied by Isasi and colleagues we know that it is very well possible to expose humans, patients, to g-profiles with a short duration maximum of 6 g in +Gz laying supine. Although microgravity countermeasure protocols are also of 30 min to 1 hour in duration, going shortly to higher peak g levels of 6 g might be more effective to prevent near weightlessness related pathologies.

In addition, the short-arm systems under study for space exploration producing a huge body g-gradient of more than 300% (Goswami et al., 2021) does not reflect the gravity exposure a body is exposed to on Earth. One might also explore the application of long-arm systems where the body g-gradient is far less.

The alternative for maintaining proper physiology is to provide chronic gravity during long duration space missions. This would be achieved generating AG by rotating the complete spacecraft, or a large part of it, as it was already proposed by Tsiolkovsky. The g-level would be continuous like on Earth (at 1 g) or, when shown by research, a lower g-level but sufficient to maintain a healthy physiology. In such a configuration the crew also does not loose valuable crew time since in a short-arm system the subject cannot perform regular tasks when spinning because of the physical restraints but also the work capacity and tolerance are reduced compared to centrifuges with larger diameters (Nyberg et al., 1966).

**FIGURE 6:**
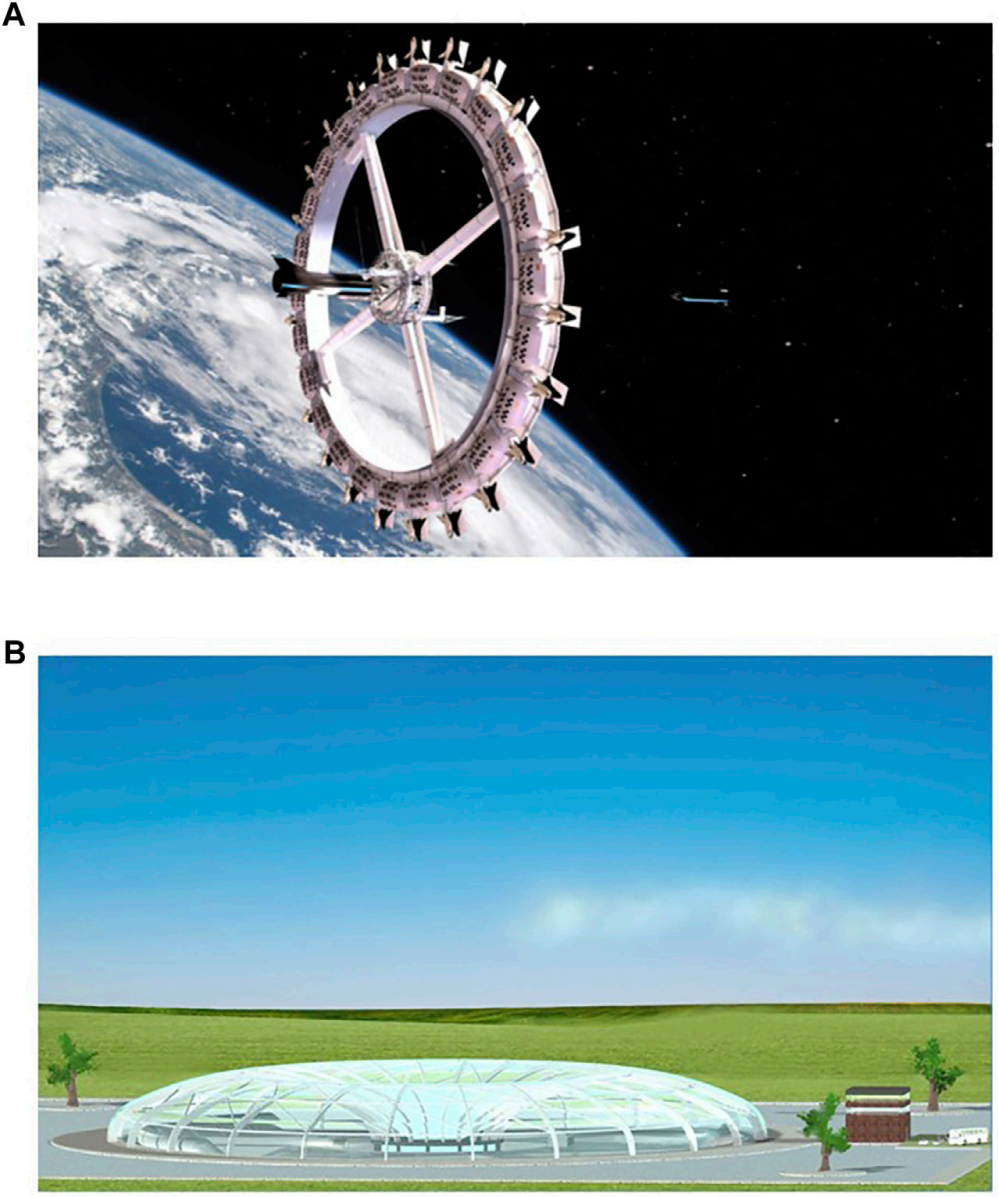
**(A)** The Voyager-class space station as proposed by Orbital Mechanics company (Fontana, CA, United States). This would be a ∼200-m diameter continuous gravity (1/3 g) space station/space hotel planned for operation in 2027. (Image courtesy: Orbital Assembly, Huntsville, AL United States)) (Spilker, 2020). **(B)** A large artificial gravity platform; the Human Hypergravity Habitat, H^3^; a proposed ground-based facility that could be used to obtain operational data regarding long duration/continuous rotation and moderate hyper-g on human physiology and psychology (van Loon et al., 2012).

Although no concrete evidence is provided by the authors, the initial response to such a solution would be that it is far too expensive or far too difficult to implement large radius centrifugation as mentioned by some scientists (Pavy-Le Traon et al., 2007; Hargens et al., 2013). However, various engineering studies clearly show that it is very well possible to assemble a rotating spacecraft which would be only 10–20% more expensive compared to a non-rotating system (Joosten, 2007). Especially with the current significant drop in launch costs, this figure might even be reduced. Also, in terms of operation and spacecraft maneuvering flexibility, several recent studies have been published (Hall, 2016; Martin et al., 2016; Chen et al., 2020; Minster et al., 2020; Spilker, 2020; Dastjerdi et al., 2021) showing the feasibility of large rotating spacecraft. We should provide the crew, and later space tourists, with a physiological level of gravity the same as we do with providing a physiological level of oxygen or food. We need to provide gravity for proper health and reduce medical related mission risks. It would be unethical to deprive a human being from gravity (van Loon et al., 2020). More research should be devoted to this subject where a ground-based model for large diameter chronic rotation could be instrumental to develop the proper in-flight configuration (van Loon et al., 2012) (Figure 6B). Having such a large ground-based facility it could also be used to explore if this could be applied for treatments on *e.g.*, obesity and research regarding ageing. There are also commercial initiatives that already incorporate chronic AG in their on-orbit designs in order to provide a suitable/healthy environment for commercial customers (Figure 6A) (Spilker, 2020).

One might consider that the current suite of short-arm centrifuges developed for space-related countermeasure research could already very well be applied to also explore the use of hypergravity in various ground-based pathologies as a spin-off of the various human space programs.

In view of the great potentiality that the use of hypergravity could have to treat different medical conditions on Earth and as countermeasure to near weightlessness related pathologies, more research is necessary in this field. In this sense, it is urgently needed to establish international collaborations to foster this field of gravity research and applications in medicine. Importantly, more basic research is needed to unravel the biological mechanisms of human centrifugation and identify the cellular and molecular bases that explain the clinical improvement in patients exposed to this kind of therapy on ground but also for developing better AG protocols for long duration spaceflights.

## Abbreviations

AG, artificial gravity; CRPS, complex regional pain syndrome; CAD, coronary artery disease, ECs, endothelial cells; GT, gravitational therapy, GTC, gravitational therapy center; HDBR, head-down tilt bed rest; HV, healthy volunteers; PAD, peripheral artery disease, RP, Raynaud´s phenomenon; SAHC, short-arm human centrifugation; SSc, systemic sclerosis.
